# Tablet-Based Wearable Patch Sensor Design for Continuous Cardiovascular System Monitoring in Postoperative Settings

**DOI:** 10.3390/bios13060615

**Published:** 2023-06-04

**Authors:** Nourelhuda Mohamed, Hyun-Seok Kim, Manal Mohamed, Kyu-Min Kang, Sung-Hoon Kim, Jae Gwan Kim

**Affiliations:** 1 Biomedical Science and Engineering Department, Gwangju Institute of Science and Technology, Gwangju 61005, Republic of Korea; nonoalhodaali@gm.gist.ac.kr (N.M.); manalalnosh@gm.gist.ac.kr (M.M.); 2 Biomedical Engineering Research Center, Asan Institute for Life Science, Asan Medical Center, Seoul 05505, Republic of Korea; hyunseok.kim@amc.seoul.kr; 3 Department of Anesthesiology and Pain Medicine, Asan Medical Center, Seoul 05505, Republic of Korea; km.kang@amc.seoul.kr

**Keywords:** cardiovascular health monitoring, patch sensor, heart and lung sounds, stethoscope, electrocardiography (ECG), analog front end (AFE), tablet-based software

## Abstract

Meticulous monitoring for cardiovascular systems is important for postoperative patients in postanesthesia or the intensive care unit. The continuous auscultation of heart and lung sounds can provide a valuable information for patient safety. Although numerous research projects have proposed the design of continuous cardiopulmonary monitoring devices, they primarily focused on the auscultation of heart and lung sounds and mostly served as screening tools. However, there is a lack of devices that could continuously display and monitor the derived cardiopulmonary parameters. This study presents a novel approach to address this need by proposing a bedside monitoring system that utilizes a lightweight and wearable patch sensor for continuous cardiovascular system monitoring. The heart and lung sounds were collected using a chest stethoscope and microphones, and a developed adaptive noise cancellation algorithm was implemented to remove the background noise corrupted with those sounds. Additionally, a short-distance ECG signal was acquired using electrodes and a high precision analog front end. A high-speed processing microcontroller was used to allow real-time data acquisition, processing, and display. A dedicated tablet-based software was developed to display the acquired signal waveforms and the processed cardiovascular parameters. A significant contribution of this work is the seamless integration of continuous auscultation and ECG signal acquisition, thereby enabling the real-time monitoring of cardiovascular parameters. The wearability and lightweight design of the system were achieved through the use of rigid–flex PCBs, which ensured patient comfort and ease of use. The system provides a high-quality signal acquisition and real-time monitoring of the cardiovascular parameters, thus proving its potential as a health monitoring tool.

## 1. Introduction

Monitoring the cardiovascular and respiratory system status of postoperative patients in postanesthesia or the intensive care unit, as well as the assessment of the results, are essential in postoperative care to prevent complications and allow immediate medical interventions to save the patient’s life [1,2]. Auscultating the heart and lung (H and L) sounds as well as measuring the electrical activity of the heart can provide valuable information to monitor and assess the patient status [3].

Auscultation is defined as ‘the art of listening to the heart and lung sounds via loudspeaker’ [4], and it can be achieved by different methods such as using chest stethoscopes, esophageal stethoscopes, or digital systems [5]. Although an esophageal stethoscope is a simple method for detecting heart and lung sounds and can provide much less noisy sounds, the esophageal catheter must be inserted into the patient’s body to detect the sounds, which limits its ability to be used for continuous monitoring [6]. On the other hand, a chest stethoscope is a simple device that consists of a chest piece with a diaphragm or bell or both, in addition to a tube and an earpiece [7]. The chest piece is attached to the patient’s chest and works as a physical amplifier to amplify the mechanical sounds detected by the diaphragm. The tube is a conductor that delivers the detected sounds to the earpiece through which the physician can hear those sounds for diagnosing purposes. The main disadvantages of the ordinary chest stethoscope are that the diagnosis mainly depends on the skills and expertise of the physician, and the acquired data cannot be stored, analyzed, or shared with other physicians [8]. The process of digitizing the stethoscope is widely adopted nowadays so that the data can be stored for further processing [9]. A digital stethoscope can be achieved by means of utilizing the same stethoscope head while replacing the acoustic transducer with microphones or other sound-detecting devices. The frequency range parameter plays a major role in selecting the appropriate microphones. Heart sounds frequencies range from 24 to 144 Hz, with a dominant frequency at 48 Hz [10], while lung sounds frequencies range from 50 to 2500 Hz, with an upper limit of 2000 Hz in abnormal cases [11,12]. Electret condenser (EC) microphones are widely used to detect H and L sounds because of their appropriate flat frequency response, small size, low cost, and high fidelity [13]. The detected signal is then amplified to a level that can be detected by a processor, and various processes such as analog-to-digital conversion and filtering can be applied [14].

Collecting sounds from the patient chest using a stethoscope makes the process prone to noise collection from the surrounding environment [15]. There are various techniques available for noise reduction, including the use of analog filters and digital filters. Among these available solutions, adaptive noise cancellation (ANC) techniques are gaining increasing attention due to their effectiveness in removing the noise corrupted with the signal [16]. ANC filters implement one of the adaptive algorithms, such as least mean square (LMS) or recursive least square (RLS), to better approximate the noise pattern and subtract it from the main signal to achieve a noise-free signal [17]. The LMS algorithm has the advantages of being simple, less complex, and requiring less memory for computation. Two signals need to be fed to the algorithm to perform the noise cancellation: the first signal is the main signal that needs to be denoised, and the second signal is the noise itself that is corrupted with the main signal; by specifying the algorithm parameters, the algorithm achieves its goal of canceling the noise by minimizing the cost function. The algorithm follows a specific equation to update values of the filter coefficients.

Electrocardiography (ECG) is one of the most popular and widely used technique to monitor and assess the cardiovascular systems’ electrical activity by using surface electrodes placed on the patient’s chest [18]. To acquire the ECG signal, various lead systems can be used such as a single-lead system, a three-lead system, and a twelve-lead ECG system. Short distance ECG measurement using a three-lead system has been proven for its effectiveness and high-quality data acquisition [19], in which three electrodes are used and placed on the chest around the heart, two electrodes form a three-lead ECG system, and the third one is the right leg drive (RLD) electrode, which is used to improve the common mode rejection ratio (CMRR) by rejecting noise and interferences from external sources [20]. After sensing the signal from the electrodes, an acquisition circuit is required to amplify the signal to a level that can be detected by the controller and filtered from the noise that may corrupt the signal. Analog front end (AFE) is a name given to the integrated IC chip that provides the ability to apply these processes. The family of ADS129x AFEs is widely used in biopotential signals acquisition systems, such as the ECG, the electroencephalography (EEG), and the electromyography (EMG), because of their integration and high precision [21]. The ADS1298 is an AFE with a high resolution, 24-bit delta–sigma (∆Σ), analog-to-digital convertors (ADCs), and low noise built in its programmable gain amplifiers (PGAs) [22] that make it a good choice to measure the ECG signal.

Nowadays, the technology of wearable biosensors has been widely adopted in the medical devices design process. It is becoming achievable, as there is a great advancement in integrated circuits designs, sensors technologies, printed electronics, and mobile-based software development [23]. A lot of studies have been conducted on wearable ECG monitoring devices to monitor and asses the cardiovascular system status, as reported in [18,24]. They are all working towards achieving the miniaturization of the device while simultaneously collecting a large number of signals, which is a significant challenge. The rigid–flex printed circuit board (PCB) is an electronic board manufacturing technique that combines the use of both rigid and flexible parts on the same board to benefit from the advantages of both and overcome the limitations of traditional rigid boards. It allows for lightweight weight, compactness, reduced package size, and the miniaturization of the developed device. It also adds to the integrity and soldering reliability of the application. Despite all these advantages, it has few applications in wearable bioelectrodes design.

Tablet-based applications for monitoring physiological parameters are currently being widely adopted due to the technological advancement and availability of tablets and smart phones [25]. Android-based software can be easily developed and shared with other mobile devices [26], which allows for the use of the developed medical device, together with the developed software, in different settings. Since these tablets contain processors, the collected data can be continuously displayed in real-time and can also be further processed and analyzed for diagnostic purposes. Although a lot of studies have used smartphones for displaying purposes, but they still lack the continuity of displaying and monitoring the derived signals parameters.

In this study, a tablet-based patch wearable sensor design that can continuously display and monitor the collected, as well as the derived cardiopulmonary parameters, is presented and can be used in the postoperative setting. The patch allows for the measurement of three main signals, i.e., the H and L sound signals and the ECG in real-time by using a modified commercial chest stethoscope head and microphones to form an electronic stethoscope using short-distance ECG measurement techniques to achieve a three-lead ECG system. An LMS algorithm was implanted to remove the background noise corrupted with the main H and L sounds, and a highly sophisticated tablet software was used to display the system output.

## 2. Materials and Methods

**Figure 1:**
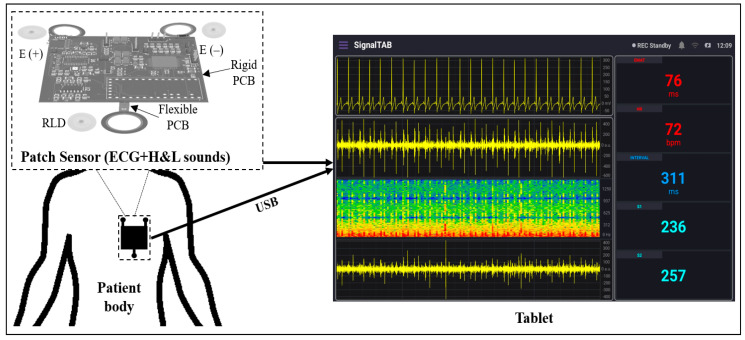
Block diagram of the overall system.

This study aimed to design a lightweight, wearable patch sensor that could be used to monitor the cardiovascular health of the patient in postoperative settings. This patch device may be used in the postanesthesia care unit (PACU) or the intensive care unit (ICU) for real-time and continuous monitoring of the H and L sounds, as well as the ECG signal, by means of a chest stethoscope and ECG dry electrodes. To build the system, a chest stethoscope head, two EC-type microphones, a Teensy 3.2 development board, and a tablet-based developed software were used. The stethoscope head was used to provide physical amplification for the heart and lung sounds, and one microphone was attached to the end of the head to pick up the detected sound. The second microphone was mounted on the surface of the system board at the same level as the first microphone to collect the background noise that might be detected by the first microphone; then, an adaptive noise canceling algorithm was implemented for noise reduction purposes. The short-distance three-lead ECG signal was collected from the chest using a high precision AFE device alongside an Ag/AgCl dry electrode. Teensy 3.2 was used as the system microcontroller to control the AFE and process the collected signals, as well as implement the adaptive noise cancellation algorithm, to reduce the background signals that corrupt the H and L sound signal. A tablet-based software was developed to visualize the collected signals and allow its recording for future use. A USB between the patch and the tablet was used for data transmission and for system power. Considering the thickness of the stethoscope head and the non-flat surface of the chest, a rigid–flex type PCB was used for the final board production, and the dry ECG electrodes, as well as an adhesive tape, were used to ensure good attachment of the patch to the patient body. Figure 1 shows the basic concept of the developed wearable patch sensor.

### 2.1. Hardware Architecture

**Figure 2:**
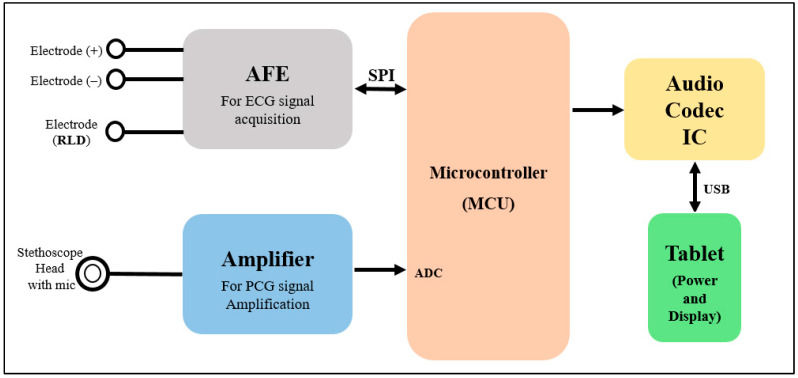
Block diagram shows the operational scheme of the proposed patch.

Figure 2 represents the basic operation concept of the developed patch sensor. The ECG signal is sensed using three dry electrodes (positive, negative, and RLD) and acquired using ADS1298 AFE. The H and L sound signal is sensed using a modified stethoscope head and a microphone, which is then fed to the amplification circuit to be amplified. Teensy 3.2 development board is used as the system main processing unit to control the ADS1298 AFE and communicate with it through SPI communication pins to acquire the ECG signal, as well as to digitize and process the detected H and L sound signals. The output signals from the Teensy are fed to the codec IC for encoding and decoding purposes. Finally, the output of the codec IC is displayed using a tablet-based software developed mainly for this study.

### 2.2. Stethoscope Head Design

**Figure 3:**
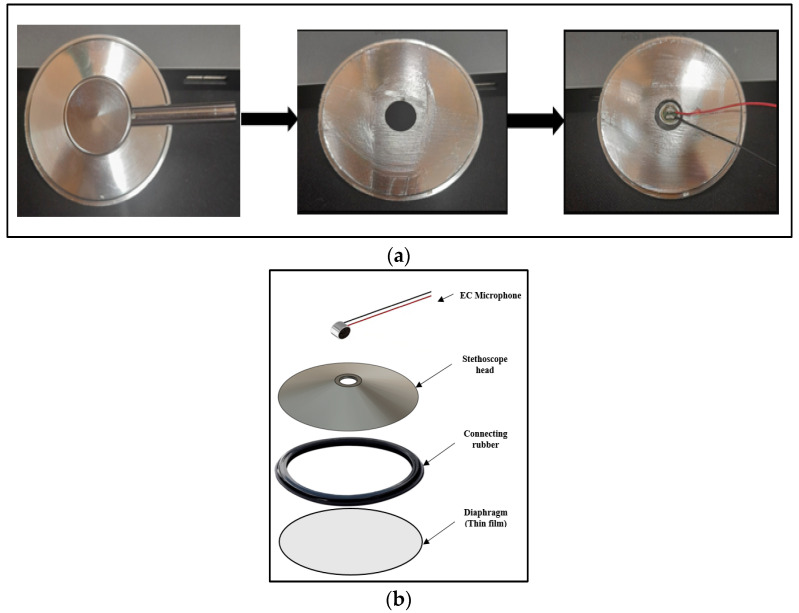
Stethoscope head design: (**a**) head modification; (**b**) head assembly.

A commercial stethoscope head was used in the patch design. To achieve the lightweight and wearability goals of the system design, some modifications were performed. The upper part of the head was trimmed so that the EC microphone could easily be fixed to the head to detect the H and L sounds. Figure 3 shows the stethoscope head modifications and assembly process.

The circular shape of EC microphones allows for good contact and connection with the stethoscope head. A POM-2738L-LW100-R analog EC microphone (PUI Audio Inc., Hangzhou, Zhejiang, China) was used in this study. It has a frequency response of 15 to 16 KHz, which is appropriate for the detection of H and L sounds; also, its light weight, high sensitivity, and high signal-to-noise ratio parameters made it a good choice for this study’s purposes.

### 2.3. Acquisition and Processing Units

The patch acquisition and processing unit consisted mainly of four parts: the H and L sounds signal acquisition part, the ECG signal acquisition part, the Teensy 3.2 development board as the system microcontroller, and the codec IC and USB part to display the system output on the developed tablet software. These circuits’ design was formulated using Altium Designer software (version 2022, Altium Limited, Chatswood, Australia), which allows for smooth designing capability, as well as flexible PCB designing and layer stacking abilities, that meet the purpose of this study.

#### 2.3.1. PCG Signal Acquisitions Circuit

**Figure 4:**
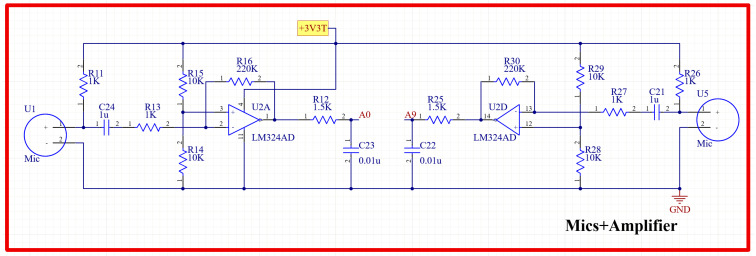
Schematic diagram of the H and L sound signal amplification circuit.

The signals picked up by the two EC microphones were fed into an amplification circuitry in order to amplify them to a suitable level that could be detected by the microcontroller. The schematic diagram of the power amplifier circuit used is provided in Figure 4. An LM324 operational amplifier (Texas Instruments, Dallas, TX, USA) was used for the amplification because of its high frequency response in the frequency range of interest of this study. The amplified signals were then fed into the microcontroller to implement the adaptive filtering algorithm and, thus, ensure a noise-free signal that could further be displayed using the tablet-developed software.

#### 2.3.2. ECG Signal Acquisition Circuit

**Figure 5:**
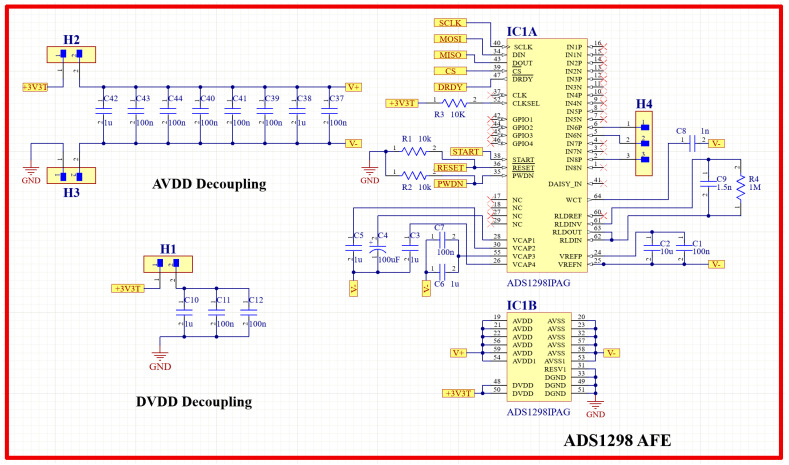
ECG acquisition circuit schematic diagram using ADS1298 analog front-end.

For the purpose of designing a wearable sensor patch, short-distance ECG signal acquisition was implemented. Other research studies have proved that a meaningful ECG signal can be acquired at a minimum electrode distance of 24 mm [27]. The family of ADS129X front-end ICs is one of the best available products to be used in this system, as they ensure high-precision measurement of the ECG signal because of their high resolution, high data rate, and high CMRR. It also has a light weight, small size, compactness, and high level of integration that results in a reduced system size. An ADS1298IPAG AFE (Texas Instruments, Dallas, TX, USA) was chosen for this design because of its high resolution. This AFE was integrated into the patch to implement a three-lead ECG measurement system. Three H124SG 24 mm disposable Ag/AgCl electrodes (Kendall, Australia) were used to pick up the ECG signal. Figure 5 shows the schematic diagram of the ECG acquisition circuit using the ADS1298 IPAG 24-bit analog front-end. This circuit communicates with the microcontroller through SPI communication so that Teensy can control the analog front-end and receive its output. The final ECG signal has been displayed using the developed tablet software.

#### 2.3.3. Patch Processing Unit

**Figure 6:**
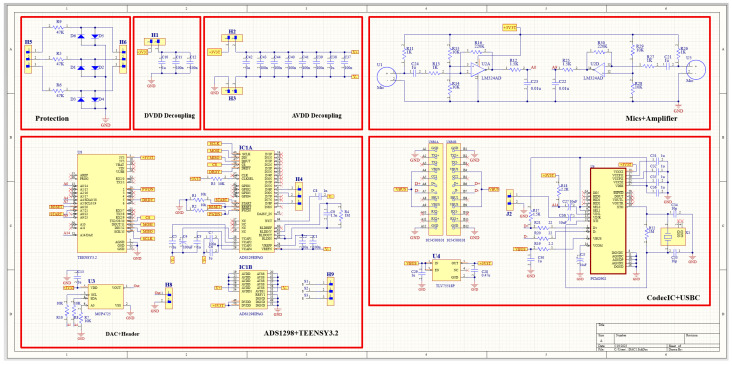
Schematic diagram of the overall system.

The schematic diagram of the overall system is presented in Figure 6. A patient protection circuit was developed using diodes and resistors to protect both the patient and the circuit from the adverse current. After acquiring both the H and L sound signals and the ECG signal, they were fed into the Teensy 3.2 development board (Adafruit, New York, NY, USA) for processing and displaying purposes. Teensy 3.2 has a processing speed of 72 MHz; it also has a lightweight and small size. The DMA feature of the Teensy 3.2 makes it best suited in this study, as data can be acquired, compressed, and transferred in real-time. The two signals from the microphones were fed into the adaptive filter algorithm that ran by the Teensy. LMS adaptive algorithm was implemented to achieve the filtering. A SIMULINK model (refer to Appendix A) was developed to simulate the real two signals that would be fed to the algorithm, and different parameters values were tested until the best parameters were achieved; then, all of the adaptive filter parameters were set, and the algorithm was implemented in the microcontroller code. To prepare the data to be visualized by the developed tablet software, a PCM2902CDB codec IC (Texas Instruments, Dallas, TX, USA) was used. It is a complete USB audio codec with a maximum sample frequency of 48 KHz. This IC includes a USB controller for ADC and DAC, as well as an HID part for three buttons, which include output volume control and an S/PDIF encoder and decoder. It also has two audio channels that can be used to encode/decode the two signals processed by the Teensy and send them to the tablet to be displayed using the developed software. A USB type C (Molex, China) was used as a connection medium between the patch and the tablet to carry the two signals for display and also to power the patch from the tablet. As this patch was used for continuous auscultation, it was better to be powered continuously from the tablet to ensure proper function.

For the purpose of programming the microcontroller, the Teensy 3.2 is a USB device, i.e., it can be programmed using USB 2.0 cable. After programming the Teensy, the USB type C connected between the tablet and the patch was used for powering the patch, and there was no longer a need for using the Teensy USB cable.

**Figure 7:**
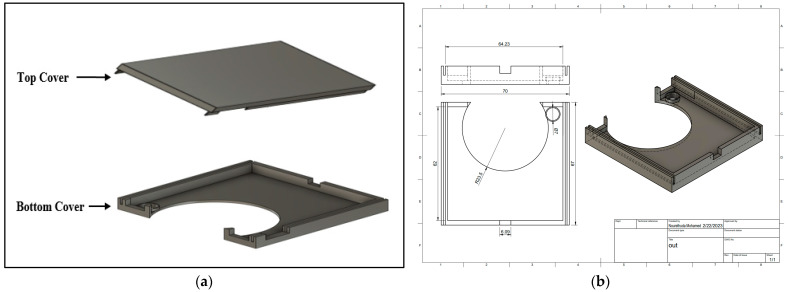
(**a**) 3D view and (**b**) drawing of the 3D-printed case.

To avoid the detachment of the flexible parts of the board while in use and to ensure the good attachment of the patch to the patient body, a high-quality 3-D printed case was designed using Fusion 360 modeling software (Autodesk, Inc., San Francisco, CA, USA) to enclose the different patch parts so that only the stethoscope head and the ECG electrodes would be visible for the user while using. Figure 7 shows the drawing and dimensions of the designed case.

### 2.4. System Software

**Figure 8:**
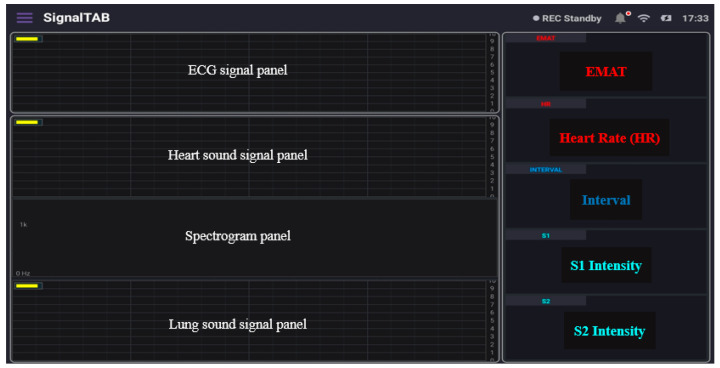
The different panels of the developed tablet software.

SignalTAB, which is an Android software developed for this study, was used to visualize the measured health signals of the system. It was written using Java programming language, and different panels were designed to display the collected signal, as well as some calculated parameters, as shown in Figure 8. This software was developed to be able to continuously display and monitor the collected and the derived cardiopulmonary parameters to ensure that all the processing would be done in real-time. A Chebyshev digital filter was used to filter out the acquired signals. The heart sound signal was band-pass filtered in the range between 25 and 100 Hz so that it would not mixed with the lung sound signal, and this made knowing the location from which the sound was coming easy to discern, and a sampling rate of 4 KHz was used to display it. The lung sound signal was band-pass filtered between 300 and 1800 Hz so that picking up all of the lung sounds would be achievable, even in case of abnormality, and its sampling frequency, together with the ECG signal, was set to be 4 KHz. The spectrogram is a very powerful feature that can be calculated to provide a detailed visualization of the sound by representing the time, frequency, and amplitude in one graph [28], and it also can be used for feature extraction for applying machine learning techniques [29]; in this study, the spectrogram of the heart sound signal was computed using the short-time Fourier transform (STFT) using 4000 samples per second and an FFT size of 2048 samples. The FFT was calculated every 2048/4000 = 512 ms, with a frequency resolution of 4000/2048 ≈ 1.95 Hz. From the collected ECG signal, the heart rate (HR) was calculated and displayed, and, from the heart sound signal, the intensities of the first heart sound signal (S1) and the second heart sound signal (S2) were calculated and displayed. In addition, the interval between S1 and S2 (INTERVAL) was calculated and displayed as well. The electromechanical activation time (EMAT) is the time interval from the onset of the Q-wave on the ECG signal to the peak of S1 [30]. This parameter is related to the cardiac contractility and provides a good indicator about the heart health, so it was also calculated and displayed in the software. The software also provides the ability to record the measurements and save the recorded data in the tablet memory for future processing.

## 3. Results

### 3.1. System Hardware

**Figure 9:**
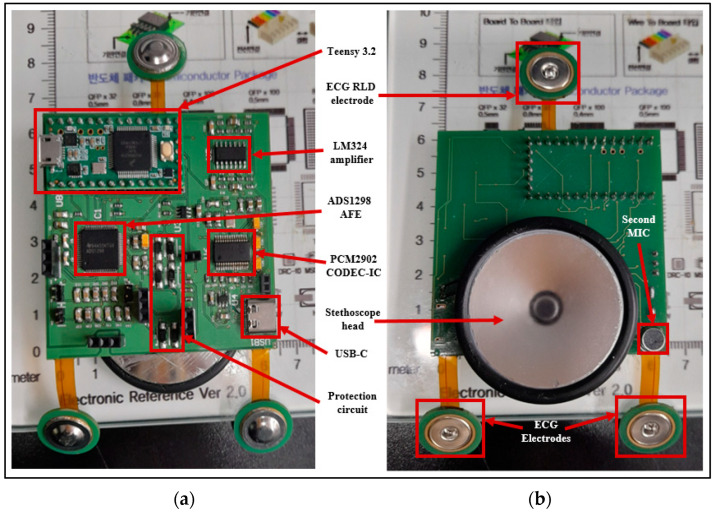
(**a**) Top view and (**b**) bottom view of the manufactured PCB board.

**Figure 10:**
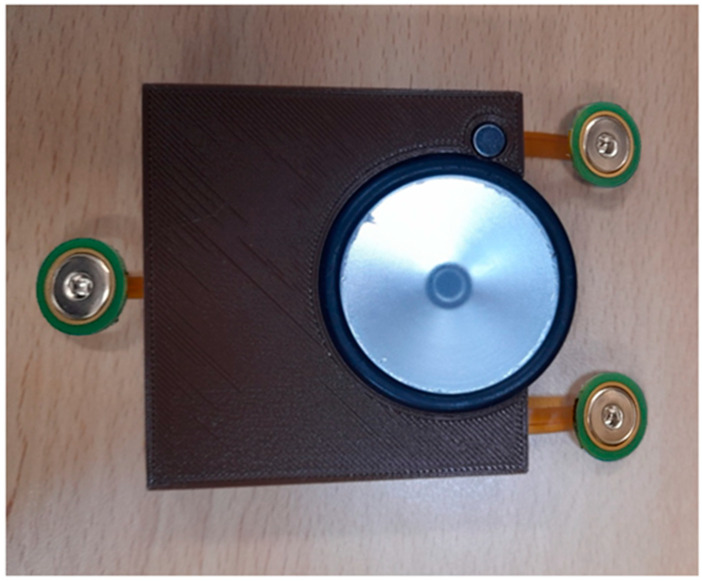
Final assembled patch sensor.

Figure 9 shows the top and bottom views of the developed patch sensor prototype. At the top, all of the signal acquisition and processing electronics were placed. In addition, the microcontroller that controls the communication between the different parts and communicates with the visualization software was placed on the top. On the bottom side, the stethoscope head attached to the microphone to detect the H and L sounds was placed. The additional microphone that captures the background noise and allows for the implementation of the ANC was also placed at the bottom to be on the same level as the first microphone. The three ECG electrodes were placed in the flexible part of the board to allow them, together with the stethoscope head, to have good contact with the patient’s chest. The prototype was enclosed in the 3D-printed case, and the finally assembled patch sensor is shown in Figure 10.

### 3.2. System Software and Experimental Results

**Figure 11:**
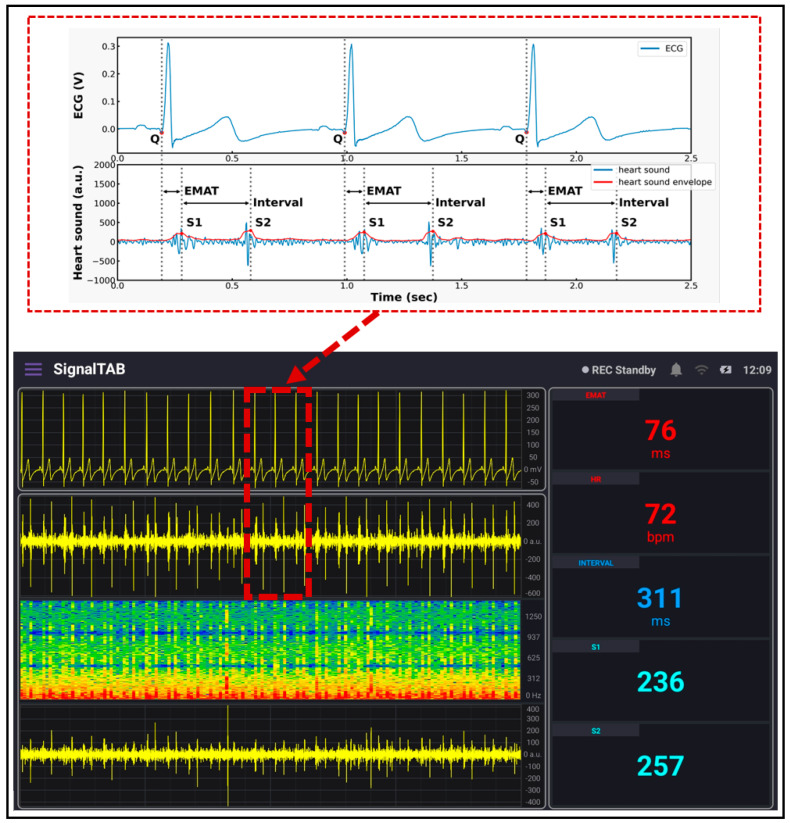
ECG/PCG signals recorded using the developed system.

The developed patch sensor was placed on the chest of a volunteer subject to evaluate its performance as a cardiovascular system health monitoring device. Since the auscultation points for the heart sounds are different from those for the lung sounds, the developed patch was placed at two different locations on the chest. First, the heart sounds and the ECG signal were recorded simultaneously by placing the proposed patch sensor on the tricuspid valve auscultation point of the subject’s chest, who was asked to hold their breath during the recording to acquire a neat and less noisy heart sound signal. A continuous and real-time measurement was obtained for 5 min. Figure 11 below shows the measured data. The upper panel in the software shows the ECG signal. Compared to the standard ECG signal, which contains the P wave, QRS complex, and T wave, the ECG signal measured using the developed patch contained all those three components with an almost similar pattern. The upper part of Figure 11 shows a detailed graph of the signal collected after being saved to the tablet. The onset of the QRS complex was marked with the letter Q. The peak-to-peak amplitude of the measured ECG was found to be 300 mV, which is in the normal range for an adult subject [31]. The second panel shows the measured heart sound signal, in which the first and second heart sounds (S1 and S2) are clearly visible. This signal pattern is similar to the standard heart sound signal measured from the tricuspid valve auscultation point. In addition, a detailed graph of the heart sound signal is shown in the upper graph with clarification on how the interval, S1, and S2 intensities were calculated. In addition, the envelope of the signal that outlines its extremes was calculated and displayed. The EMAT value, which is the time difference between the onset of the Q signal and that of the first heart sound, was calculated from the acquired data, and it was found to be 76 ms. This value is physiologically reasonable for healthy adults [32], and it ensures the synchrony of the measured data. The heart rate value calculated was found to be 72 beats-per-minute, which is also within the normal range for healthy adults.

**Figure 12:**
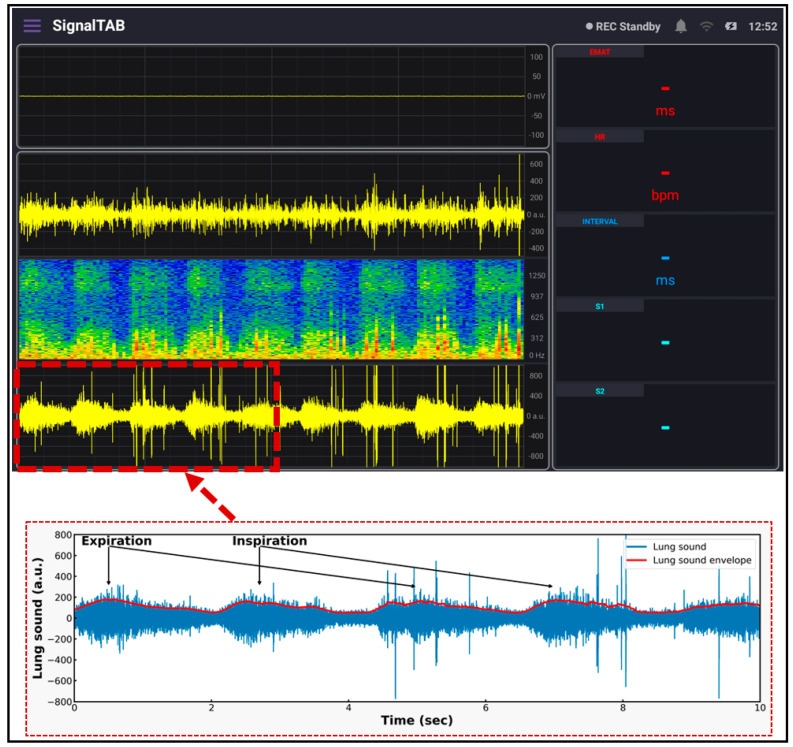
Lung sound signal recorded using the developed system.

For measuring the lung sound, the developed patch sensor was placed in the anterior lung auscultation point in the first intercostal space of the subject’s chest. The subject was asked to breathe deeply so that the inspiration and expiration sounds could clearly be heard by the microphone and recorded. The measurement lasted for 3 min, and the results are shown in Figure 12. The inspiration and expiration signals are clearly visible in the lung sound panel, with some spikes related to heart sound and subject movements. A detailed graph showing the signal after being saved is shown in the lower part of the figure. The inspiration and expiration time periods are clearly annotated, and the envelope of the signal was calculated and displayed. Compared to the standard lung sound signal from the same auscultation point, the developed patch signal had the same pattern, and the inspiration sound was much louder than the expiration signal, which is consistent among adults [33].

## 4. Discussion

Auscultation of the heart and lung sounds using a chest stethoscope is a popular technique used to check the health of the heart and to diagnose the problems related to the heart valves [34]. Stethoscopes constitute a widely available, cheap, simple, and highly trusted technique used by physicians to auscultate the H and L sounds; however, they require a highly skilled and experienced physician to have a good diagnosis [35]. The ECG, on the other hand, is a common, painless, and effective test used to monitor the cardiovascular system. It provides good insights about the heart conditions and quickly detects heart-related problems [36]. This study presents a bedside monitoring system design that utilizes a lightweight and wearable patch sensor. Three main signals, i.e., the heart sound signal, lung sound signal, and ECG signal, were collected using the proposed device to allow for the real-time and continuous monitoring of the cardiovascular system status of patients who undergo cardiac operations [37].

Advancements in electronics manufacturing technologies have made it possible to achieve a reduced, miniaturized, and high-quality data acquisition device [38]. Various technologies can be used for the device boards manufacturing, such as rigid board design, flexible board design, and the combination of both, i.e., rigid–flex board designs. This variability allows for the robustness, reliability, and integrity used in demanding applications. Rigid–flex PCBs allow for the flexibility of manufacturing the board to fit into the device so that it can fit confined or smaller areas. It also has fewer numbers of interconnections, reduced circuitry failure, and a relatively long life-span. In this type of board, the rigid electronic components are placed in the rigid part of the board so that soldering reliability is possible, while the flexible parts are used as connectors between the rigid parts to conduct the signals [39]. Currently, these types of flexible and rigid–flex boards are widely adopted in the design of wearable biomedical devices to allow for flexibility, compactness, and small device dimensions. The developed patch was based on rigid–flex PCBs to allow for robust contact with the curved chest surface, as well as wearability for long time, to achieve continuous monitoring [40]. It also collected the ambient noise that might be detected with H and L sounds and applied an ANC technique to remove this noise to have a high-quality signal acquisition [41]. Using MATLAB and SIMULINK [42], the two main parameters of the ANC–LMS technique were determined and fed to the algorithm that was implemented in real-time using a high speed processor. The signal-to-noise ratio (SNR) measure was calculated before and after the application of the filter in the SIMULINK model, and it was found to have improved by approximately 79%.

ECG, together with PCG, were first validated by placing the developed patch sensor on the chest of a volunteer subject. The acquired signals were found to be of good quality, as shown in the ECG graph, the P, QRS, and T components of the ECG signal are clearly visible; in addition, in the PCG graph, the S1 and S2 signals were well recorded and demonstrated. As a second step of validation, the developed patch sensor was placed at a specific lung auscultation point, and the lung sound was recorded. The inspiration and expiration sound signals were clearly visible and distinguishable in the lung sound graph. The spectrogram for both heart sound and lung sound signals was also displayed using the software. Other parameters calculated from the measured signals were also continuously displayed.

A comparison between the current study and previous similar developed designs is conducted and presented in Table 1. Although these studies proposed a design to monitor the cardiopulmonary system, they primarily focused on the auscultation of heart and lung sounds, which mostly served as screening tools. This study presents a novel approach to continuously display and monitor the derived cardiopulmonary parameters by proposing a bedside monitoring system that utilizes a lightweight and wearable patch sensor for continuous cardiovascular system monitoring. To our knowledge, one study measured the same three signals as this study, but it did not apply filtering to the measured H and L sound signals. This study applied an adaptive cancelling denoising algorithm to the signal in real-time while data was being collected. The other studies were either measuring the ECG only or the H and L sound signals only. The resolution, as well as the data sampling rate, were very high compared to the other systems. Only one previous study was based on a rigid–flex PCB design, which has a lot of advantages over traditional rigid boards, including flexibility to attach to the body, compactness, and small device dimensions. In addition, the developed patch has a small size, with dimensions of 61 × 62 mm^2^ and a light weight of 50 g compared to other devices having the same functionally. The data collected was displayed using a computer tablet, which ensured continuous data acquisition and better visualization in real-time, as well as storage and further processing of the data in the tablet itself, so there was no need to further send the data to another PC.

## 5. Conclusions

In this study, a prototype for measuring the heart sound, the lung sound, and the ECG signals from a patient in a postoperative setting was designed. The developed patch sensor has a light weight and can easily be worn by the patient without complications to achieve continuous auscultation and ECG signal recording to monitor the cardiovascular system health status. The auscultation was achieved by means of using the chest stethoscope head and a microphone. A second microphone was used to collect the background noise, and an ANC algorithm was implemented to achieve noise-free signals. The ECG signal was measured using a short-distance ECG measurement by using ECG dry electrodes and ECG AFEs. A high-speed microcontroller to control and process the data and to implement the ANC algorithm was used. Finally, the collected signals were displayed using tablet-based software that was developed especially for this study purpose. The small, lightweight, and high-quality data acquisition properties of the developed patch sensor ensure its wearability and effectiveness as a cardiovascular health monitoring device. In addition, its flexibility ensures good contact with the curved surface of the patient’s chest and their comfortability, and the patch stability is supported by the ECG electrodes and the use of adhesive tapes.

## Figures and Tables

**Table 1:** A comparison between the developed patch and other similar developed systems.

Specifications		[19]	[3]	[43]	[23]	[44]	[45]	[46]	This System
Acquired Signals		ECG, H and L Sounds, IP	HS, PPG	Temperature, BP, ECG	H and L Sounds	ECG	ECG	ECG, HS, Physical Activity	ECG, H and L Sounds
**ECG**	Leads	one	-	three	-	three	three	one	one
	Electrode Distance	short		short		short	normal	normal	short
	Electrode Type	active dry		active dry		active dry	floating	active dry	active dry
	Sampling Rate (KHz)	1		0.125		0.256	-	-	4
	Resolution (bit)	16		-		12	-	-	24
**Heart sound**	Sensor	MEMS mic	MEMS mic	-	mic	-	-	piezoelectric	EC–mic
	Sampling Rate (KHz)	10	-		0.25–2			-	4
	Denoising Tech.	-	-		-			-	ANC–LMS
**Lung Sound**	Sensor	MEMS mic	-	-	mic	-	-	-	EC–mic
	Sampling Rate (KHz)	1			250–2000				4
	Denoising Tech.	-			-				ANC–LMS
**PCB**	Type	rigid	rigid	flexible	rigid–flex	rigid	-	-	rigid–flex
	Dimensions (mm)	70 × 60	98 × 40	-	-	30 × 30	102 × 102	-	61 × 62
	Weight (g)	-	85	<0.5	-	12 g	-	-	50
**Wearability**		wearable	wearable	wearable	wearable	wearable	wearable	wearable	wearable
**Displaying Method**		-	smartphone	smartphone	smartphone	PC and smartphone	PC or smartphone	notebook	computer tablet
**Monitoring Type**		-	long-term	long-term	long-term	long-term	continuous	remote	continuous
**Application Field**		sleep monitoring	-	-	-	-	-	Home-based	PACU/ICU

**Figure A1:**
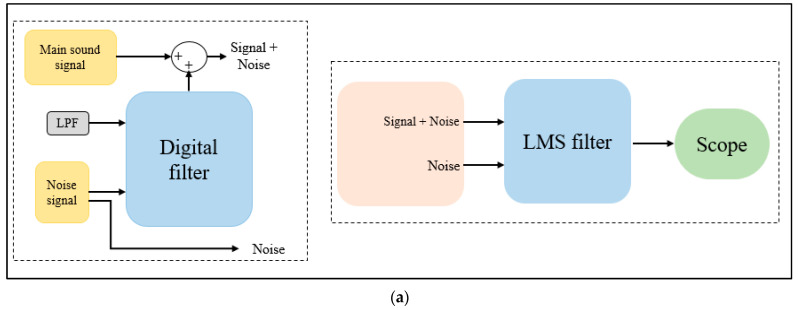

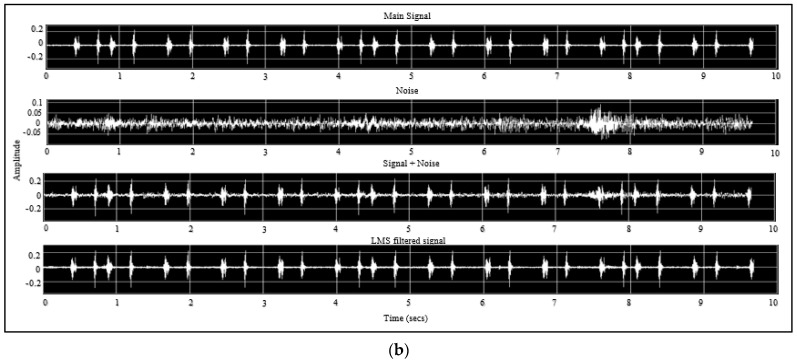
SIMULINK model (**a**) design and (**b**) results.

## Data Availability

The data presented in this study are available upon request from the corresponding author. The data are not publicly available, because they are still under collection.

## Appendix A

To determine the LMS filter parameter, a SIMULINK model was developed using MATLAB R2021a. First, simulated data was generated using a digital filter that combined the main heart and lung sound signal and a recorded noise signal. The two signals were fed to the model as WAV files. Then, a block model was designed to simulate the application of an LMS algorithm using the built-in MATLAB LMS function. The step size and the filter length are the main parameters that needed to be determined for applying the LMS algorithm [47]; accordingly, various step size and filter length values were tested to figure out the their best values. Figure A1a shows the block diagram of the developed model, and Figure A1b shows the SIMULINK model results.
